# Drought and child undernutrition in Ethiopia: A longitudinal path analysis

**DOI:** 10.1371/journal.pone.0217821

**Published:** 2019-06-17

**Authors:** Bezawit Adugna Bahru, Christine Bosch, Regina Birner, Manfred Zeller

**Affiliations:** 1 Rural Development Theory and Policy, Institute of Agricultural Sciences in the Tropics (Hans-Ruthenberg-Institute), University of Hohenheim, Stuttgart, Germany; 2 College of Agriculture and Veterinary Medicine, Jimma University, Jimma, Ethiopia; 3 Social and Institutional Change in Agricultural Development, Institute of Agricultural Sciences in the Tropics (Hans-Ruthenberg-Institute), University of Hohenheim, Stuttgart, Germany; University of Dhaka, BANGLADESH

## Abstract

**Background:**

The increase in the frequency of extreme events due to climate change poses a serious challenge to achieving the Sustainable Development Goal 2 of ending hunger by 2030. While evidence exists on the impact of drought on under-five children, its effect during late childhood and early adolescence remains less investigated.

**Objective:**

This study estimates the impact of concurrent and long-term exposure to drought on linear growth during late childhood and early adolescence.

**Methods:**

Four rounds (2002–2013) of data from the young lives Cohort Study dataset (n = 2000) was used. The associations of concurrent and long-term exposure to drought and Height-for-age z-score was analysed using structural equation modelling techniques. The study also explored the mediating role of interim period growth in the association of early exposure to drought and undernutrition at later age and the role of the Productive Safety Net Program in buffering the impact of drought on child nutrition.

**Results:**

Results show that both concurrent and long-term exposure to drought was negatively associated with Height-for-age z-score (*p* < 0.001). Exposure to drought at age 5, 8, and 12 years is associated with lower Height-for-age z- score at age 5, 8, and 12 years respectively. Exposure to drought at age 5 years was also negatively associated with Height-for-age z-score at age 12 years (*p* < 0.001). This association was mainly indirect (89%) and mediated through reduced child growth in subsequent years. Participation in productive safety net program by drought-affected children reduces but does not completely offset the negative effects of drought on Height-for-age z-score (*p* < 0.1). Moreover, girls were more likely to suffer poor growth than boys.

**Conclusion:**

Drought exposure after the 1,000 days window could have a lasting impact on child growth. Given the importance of this period for child physical and mental development, children beyond the 1,000 days window should also be a focus of disaster relief programs.

## Introduction

Climate change represents a serious challenge to meet the Sustainable Development Goal 2−*’end hunger*, *achieve food security and improved nutrition and promote sustainable agriculture*’ by 2030 and leaves billions at risk of food insecurity, undernutrition, and illnesses [1]. It will lead to a more frequent occurrence of droughts, floods, cyclones, and heat waves [2]. In low and middle-income countries, such events are often associated with a decrease in agricultural production, a decrease in consumption, food insecurity, and child undernutrition [3–7].

Undernutrition is one among the top five adverse impacts of climate change [8]. Globally, over 23% of under 5 children are stunted and 7.4% are wasted [9]. Although the global figure has been declining over the past decades, progress has been slow in sub-Saharan Africa (SSA). It is also evident that climate change will further decelerate this temporal decrease and may contribute to an additional 10% increase in the prevalence of malnutrition in SSA [10]. Therefore, generating evidence from longitudinal data on a range of factors (agricultural, environmental, socioeconomic, and health) could contribute to formulate appropriate policies and programs [8] and to reorient development and humanitarian assistance [11] to tackle the negative impacts of climate change.

Notwithstanding the recent increase in the number of studies that explored the impact of climate change on undernutrition, evidence remains scant. It draws on a few heterogeneous studies that have methodological shortcomings [8]. Most of these studies emphasized the impact of a change in temperature and precipitation at country or regional level, and overlook climatic variations within country or region. Evidence is also divided as to whether extreme events affect child nutrition and by age group, geographic location, the types of event and anthropometric indices considered, and analytical approaches [3–5, 12, 13]. More importantly, while evidence exists on the impact of such events on under-five children, there remains a significant knowledge gap about their impact on nutritional status during late childhood and early adolescence. Evidence shows that this period is the second window of opportunity, next to early childhood, for “catch up growth” and to break the intergenerational cycle of undernutrition [14–17]. Undernutrition during these periods may negatively effect on schooling, cognition, health during adulthood, and earnings [17–19]. Therefore, given the importance of this critical period in determining long term welfare, examining the impact of climate extremes might be an important contribution to our understanding of the process of human capital formation [13, 20].

Thus, the main objective of the study is to estimate the effect of early exposure to drought on linear growth during late childhood and early adolescence. In so doing, it underscores the extent of children's vulnerability to the vagaries of nature and the pressing need to develop policies and programs against climate extremes in low and middle-income settings. In addition, it explores the role of the Ethiopian Productive Safety Nets Program (PSNP) in buffering the effect of drought.

To do so, it drew on four rounds of data on 2,000 children spanning over 11 years, 2002 to 2013, from the YL cohort study dataset and apply a path model to investigate the association of concurrent and long-term exposure to drought with Height-for-age z-score (HAZ) of 5 to 12-year-old children. The use of a path model allows analysing the relationship between explanatory and dependent variables at the same time [21]. It also allows the decomposition of correlations among variables and hence a better interpretation of results and a clear picture of how one variable affects another [21]. Moreover, unlike other studies from the YL dataset [3–5, 13], this study used more rounds of data and conducted a path analysis to investigate the impact of exposure to drought at an early age on nutritional status at later age and the role of interim growth along this pathway.

## Conceptual framework

**Fig 1:**
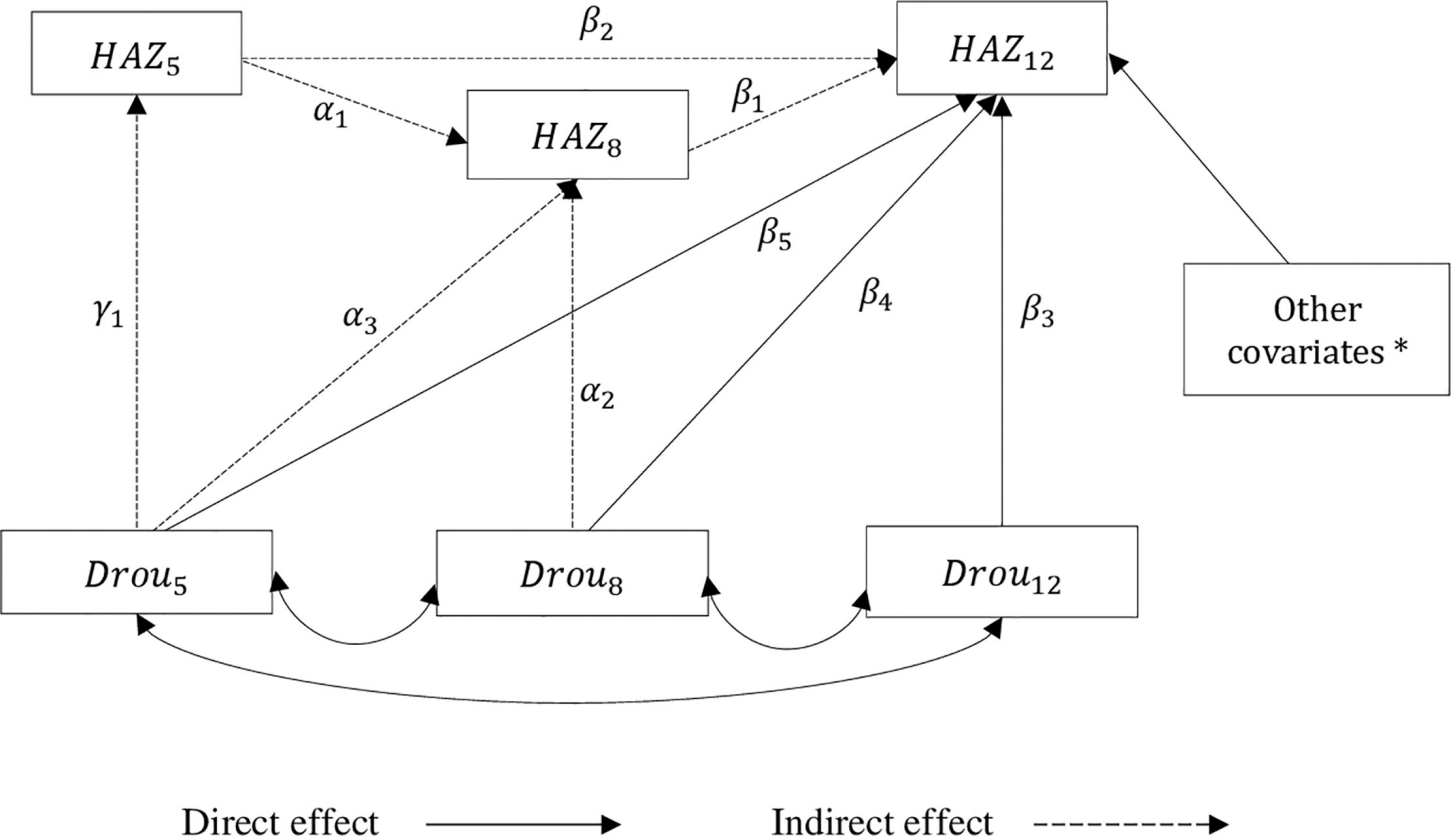
Path model of the association of drought and nutritional status at different stages of the child's development.

Previous studies have reported a negative impact of drought on chronic undernutrition among children [3–5, 12, 13]. Moreover, studies have shown the negative impacts of climatic shocks during childhood on nutritional status during adolescence [22], schooling, and adult health [23]. Exposure to climatic shocks during early childhood reduces child growth potential in growing up to subsequent years (i.e. catch up and growth faltering). Although much of the focus on the long-term impact of drought on child undernutrition has been on under-five children, exposure to drought after the 1,000 days window might have a long-lasting impact that transcends from its effect on the concurrent period to affecting nutritional status at later age. That means exposure to drought at age 5 years might also affect nutrition at age 5 years which further reduces child growth potential in growing up to the subsequent growth period (age 8 and 12 years). Similarly, exposure to drought at age 8 years might affect nutritional status at age 8 years which further reduces child growth potential in growing up to age 12. Based on that a path model summarizing the direct and indirect association of exposure to drought at age 5, 8, and 12 years (*Drou*_5_, *Drou*_8_, and *Drou*_12_ respectively) and HAZ score at age 5, 8, and 12 years (*HAZ*_5_, *HAZ*_8_, and *HAZ*_12_ respectively) was formulated. The path diagram in Fig 1 summarizes this relationship (Fig 1).

*HAZ*_5_, *HAZ*_8_, and *HAZ*_12_ refer to Height-for-age z-score at age 5, 8, and 12 years respectively. *Drou*_5_, *Drou*_8_, and *Drou*_12_ stand for drought exposure at age 5, 8, and 12 years respectively. *γ*_1_, *β*_1_, *β*_2_, *α*_1_, *α*_2_, and *α*_3_ indicate regression estimates of indirect effects. *β*_3_, *β*_4_, and *β*_5_ are regression estimates of indirect effects. * refers to the final model adjusted for covariates that are identified as important predictors of child nutrition by previous literature.

In this study, *HAZ*_12_ is considered as the main outcome of interest. The solid arrow represents a direct effect- due factors other than interim growth. The dashed arrows represent an indirect effect. This occurs when a reduction in child growth in concurrent periods due to drought reduce child growth potential in growing up to subsequent years. *Drou*_5_ might have an impact of reducing child growth directly during the same year it has occurred (coefficient for *γ*_1_) and later in life (coefficient for *α*_3_ and *β*_5_). It might also have an indirect effect on child growth at age 12 years by reducing growth potential of the child in growing up to 8 years (coefficient for *γ*_1_*α_1_) which in turn might influence the growth of the child in growing up to 12 years (coefficient for *γ*_1_*α_1_*β_1_). Similarly, *Drou*_8_ might also directly affect child linear growth in the same year (coefficient for *β*_1_) and at age 12 years (coefficient for *α*_2_ & *β*_4_). It might also affect *HAZ*_12_ indirectly through its effect in reducing child growth potential in growing up to 12 years (coefficient for *α*_2_ * *β*_4_). Moreover, *HAZ*_8_ which is a cumulative outcome of exposure to drought at age 5 and 8 years and other determinants of child nutrition might affect *HAZ*_12_ (*β*_1_). *HAZ*_12_ might also be directly affected by *Drou*_12_ (*β*_3_). Mathematical expression and details of the path model can be found S1 (S1 File).

The aforementioned impact might come about through different pathways―agriculture, environment, and income. The agricultural pathway operates through reducing crop production, increasing food prices and reducing access to food and nutrients[8, 24–26] which in turn causes household food insecurity [27, 28] and persistent reduction in food consumption [29, 30]. The environmental pathway reduces HAZ via the reduction in the availability and quality of drinking water and hence via an increase in the occurrence of diseases such as diarrhoea, fever, and acute respiratory infections [8, 31–33] which in turn affects the absorption of nutrients contained in food [34]. The income pathways operate through increasing the opportunity cost of child health expenditure and hence reducing investment in child health, reducing time devoted to child care, and introducing/increasing time burden for children [31, 35]. In addition to the nutritional status at different stages of child development and exposure to drought, out model adjusts for covariates at the child, household, and community level that might affect national status. These variables include maternal education, age, household socioeconomic status, child illness, household food security status, diet diversity score, access to health care, and PSNP enrolment (Fig 1).

## Materials and methods

### Data

This study used four rounds of the Young Lives (YL) cohort study dataset in Ethiopia. YL is a prospective cohort study designed to follow younger cohorts (n = 2,000) and older cohorts (n = 1,000) of children in Ethiopia, India, Peru, and Vietnam. This paper is based on four rounds of data from the younger cohort study in Ethiopia. The Ethiopian sample children were first enrolled in 2002 and the second, third, and fourth rounds of the survey were carried out in 2006, 2009, and 2013, respectively. YL used a multistage stage sampling technique to collect data in 20 sentinel sites (clusters). In the first stage, four regional states (Amhara, Oromia, SNNPR, and Tigray) and one administrative city (Addis Ababa) were purposively selected. In the second stage, 3–5 Woredas (districts) were selected from each regional states and the administrative city. The sample represents diverse social, geographic and demographic groups, however, Woredas experiencing food deficit were oversampled. In the third stage, one or more Kebeles (the smallest administrative unit in Ethiopia) were selected from each sample Woreda and data from a randomly selected sample of 100 YC and 50 OC children were gathered. Children were traced in each subsequent round and only 2.2% of the YC and 8.4% of the OC children were not available for follow up at round 4.

### Ethical approval

YL obtained ethical clearance from the University of Oxford Ethics Committee and the Ethiopian Public Health Institute’s institutional review board. Collective consent from the communities and informed consent from children, parents and/or guardian was obtained before the actual data collection. Details on methodology are available at http://www.younglives.org.uk.

### Measurements

#### Height-for-age z-score

Anthropometry of each child was measured by trained data collectors using the World Health Organization’s (WHO) standardized procedures [36]. Height was measured to the nearest 1mm using length board and stadiometer. Weight was measured to the nearest 0.1 kg using a calibrated digital balance (Soehnle 7831, Germany). For the present study, sex and age-adjusted HAZ was computed using the WHO standards [37, 38]. Children with implausible values of HAZ (below –6 or above +6) [36] and missing values of HAZ in two or more rounds of the survey were excluded from the analysis. For children with only one missing value, multiple imputations using chained equations was done in Stata [39] and compared the estimates with complete case analyses. No major difference in the direction of the associations, beta coefficients, and p-values was observed between the imputed and complete case analysis (S1 Table).

#### Drought

Drought was measured using a single-item question, which reads “Now I am going to ask you about the most important events and changes that have happened and negatively affected your household economy. Has drought happened since our last visit?” The response is a dichotomous variable that takes a value of “1” if the household reported “Yes” and “0” for a “No” response.

#### Dietary diversity score

YL used a 24-hour dietary recall questionnaire to gather information on child dietary diversity. Child’s consumption of one or more different variety of foods into 11 food groups was aggregated according to the Food and Agriculture Organization’s guideline [40]. Dietary Diversity Score (DDS) is then generated by summing up the number of food groups consumed.

#### Food insecurity

YL used the Household Food Insecurity Access Scale (HFIAS) to measure the prevalence of food insecurity in the household where the child resides. The HFIAS measurement score was computed following Cost et al. [41] and households were classified as severely food insecure, moderately food secure, mildly food insecure, and food secure [41]. Households were further categorized as food secure (coded as 0) if households were food secure and food insecure (coded as 1) if households were severely, moderately, or mildly food insecure.

#### Wealth index

Wealth index was computed using principal component analysis. Items used in the construction of the index include ownership of television, radio, car, motor, bicycle, landline phone, mobile phone, refrigerator, table, chair, sofa, and bedstead; the number of rooms per household member; the quality of the toilet, drinking water, cooking material, floor, roof, and wall in the household; and access to electricity. Correlation, internal consistency, and reliability of the items were checked. Items with low correlation with the rest of the items were excluded and Cronbach’s alpha value of >0.7 was obtained [42]. All variables were standardized into dummy responses and a covariance matrix was used to obtain weights of principal components followed by Bartlett’s and KMO tests of homogeneity of variance across samples (*p* = 0.000 & KMO > 0.8) [43]. After computing the wealth index, households were classified into wealth tertile as low (1), medium (2), and high (3).

#### Other covariates

Child age, sex, nutritional status of the child at round 1, child’s general health status, and dietary diversity were included as child level characteristics. Child age was measured in months and both linear and quadratic specifications were used to account for the non-linear growth of a child with age. Child sex was treated as a dichotomous variable that takes the value of “0” for a male and “1” for a female child. Among the household level covariates, maternal education was included as a categorical variable that takes a value of 0–4 if the mother had no, informal, primary, secondary, and higher education, respectively. The dependency ratio was computed as the number of non-working age members (0–12 years & >60 years) divided by the number of working age members (13–60 years) multiplied by 100. Participation in the PSNP was included as a dummy variable that takes the value of “1” if the household is a participant and “0” otherwise. With regard to community-level covariates, residence was included as a dichotomous variable that takes the value of “1” if the child lives in a rural locality and “0” otherwise. Access to a public health facility was also included as a dichotomous variable that takes the value of “1” if the child lives in a community where there is a public health facility and “0” otherwise.

### Statistical data analysis

All analyses were done using Stata version 15 [39] and a probability level of 0.05 was used to consider results as significant. Children who were not present in two and more rounds of the survey (n = 327(5.45%)) and children with implausible values of HAZ (n = 5(0.08%)) were excluded from the analysis. For the rest of the sampled children, missing values were imputed using multiple imputations with chained equation and 20 replications in Stata. The imputed missing values include child age (n = 33), HAZ score (n = 83), DDS (n = 27), maternal education (n = 648), wealth index (n = 194), dependency ratio (n = 29), experience of drought (n = 28), PSNP participation (n = 23), child health (n = 28), and food insecurity (n = 4). No major difference in the sign and significance of coefficients was observed when comparing estimates of imputed and complete case analysis (results are available upon request). A structural equation model with the full information maximum likelihood (FEML) estimation approach was done using the ‘*sem’* command in Stata 15. The overall fit of the models was assessed by the comparative fit index (CFI), Root Mean Squared Error of Approximation (RMSEA) and Standard Root Mean residual (SRMR). The parsimony index of the model was also assessed using Akaike’s information criterion (AIC)[44, 45]. Moreover, the total, direct, and indirect effects of drought on linear growth was also assessed. For robustness check, the data was fitted into ordinary least square regression, instrumental variable regression on the pooled and panel data structures, and child fixed effects. No major difference was found in the sign and significance of coefficients except a slight change in the magnitude of coefficients (S1 Table).

## Results

### Socio-demographic and economic characteristics

Table 1 shows the summary statistics of the variables used in the analysis. 1,911 children (53% boys and 47% girls) were included in the main analysis (Table 1). 46% of the sample reported the experience of drought in one or more rounds of the survey. Hereafter, we will refer to them as drought and non-drought children respectively. Significant differences in the HAZ score and other child, household, and community level characteristics were observed between drought and non-drought children. In all survey rounds, drought affected children had significantly lower HAZ scores than their non-drought counterparts (*p* < 0.001). Nevertheless, for both groups, the mean HAZ score was negative in all rounds of the survey and showed a slight improvement in all rounds of the survey over time. Drought children had a lower DDS (5.7±1.3) than non-drought children (6 ±1.4). No significant difference in general health status was observed between the two groups (*p* = 0.42).

**Table 1:** Socio-demographic characteristics, Young Lives data, Ethiopia.

	Experience of drought		P-value^1^
**Variables**	No (n = 988)	Yes (n = 838)	
**Sex (female)**	462 (46.8)	400 (47.7)	0.68
**Age (months), median (IQR**^2^**)**	145.5 (143, 149)	146.0 (142, 149)	0.19
**HAZ**^3^**, mean (SD**^4^**)**	-1.5 (-2.6, -0.4)	-1.8 (-2.9, -0.5)	0.01
Round 2	-1.3 (-2.0, -0.5)	-1.7 (-2.4, -1.0)	<0.001
Round 3	-1.0 (-1.7, -0.3)	-1.4 (-2.1, -0.8)	<0.001
Round 4	-1.2 (-1.9, -0.6)	-1.7 (-2.3, -1.1)	<0.001
**DDS**^5^**, mean (SD**^4^**)**	6.0 (1.4)	5.7 (1.3)	<0.001
**General child health status**			
Poor	50 (5.0)	36 (4.2)	0.74
Average	118 (11.7)	90 (10.6)	
Good	355 (35.2)	305 (35.8)	
Very good	485 (48.1)	420 (49.4)	
**Household food insecurity**			
Food Secure	140 (13.9)	98 (11.5)	<0.001
Mildly Food Insecure	151 (15.0)	77 (9.0)	
Moderately Food Insecure	598 (59.2)	564 (66.2)	
Severely Food Insecure	121 (12.0)	113 (13.3)	
**Wealth index (tertile)**			
Low SES6	174 (17.2)	448 (52.6)	<0.001
Medium SES6	290 (28.7)	334 (39.2)	
High SES6	545 (54.0)	70 (8.2)	
**Participation in PSNP**^7^	96 (9.5)	249 (29.2)	<0.001
**Maternal education**			
No education	283 (34.2)	419 (54.8)	<0.001
Informal education	99 (12.0)	125 (16.3)	
Primary education	269 (32.5)	204 (26.7)	<0.001
Secondary and above education	177 (21.4)	17 (2.2)	
**Dependency ratio, median (IQR**^2^**)**	0.3 (0.1, 0.5)	0.4 (0.3, 0.8)	
**Residence (rural)**	353 (35.0)	746 (87.6)	<0.001
**Health facility in the community**	662 (76.6)	323 (41.0)	<0.001

1. Result of Students t-test, Fisher's exact test, or Pearson chi-square test of H_o_ = no difference in the respective characteristics of children who are exposed to drought and who are not.

2. IQR = interquartile range.

3. HAZ = Height-for-age standard score.

4. SD = standard deviation.

5. DDS = dietary diversity score.

6. SES = socio-economic status.

7. PSNP = Productive Safety Net Program.

Considering household-level covariates, the highest prevalence of food insecurity was reported by drought exposed households (*p* < 0.001). Drought affected households also account for a higher proportion of households in the lowest wealth quantile. PSNP enrolment was higher among those who were exposed to drought (29%) than those who were not (9.5%) (*p* < 0.001). In both groups, a significant proportion of mothers had no formal education; however, mothers of drought exposed children had a significantly lower level of formal education (*p* < 0.001). The proportion of dependent household members was higher among those who had experienced drought (*p* < 0.001). Moreover, a significantly higher proportion of drought-affected children (87%) lived in rural areas (*p* < 0.001).

### Patterns of drought over time

**Fig 2:**
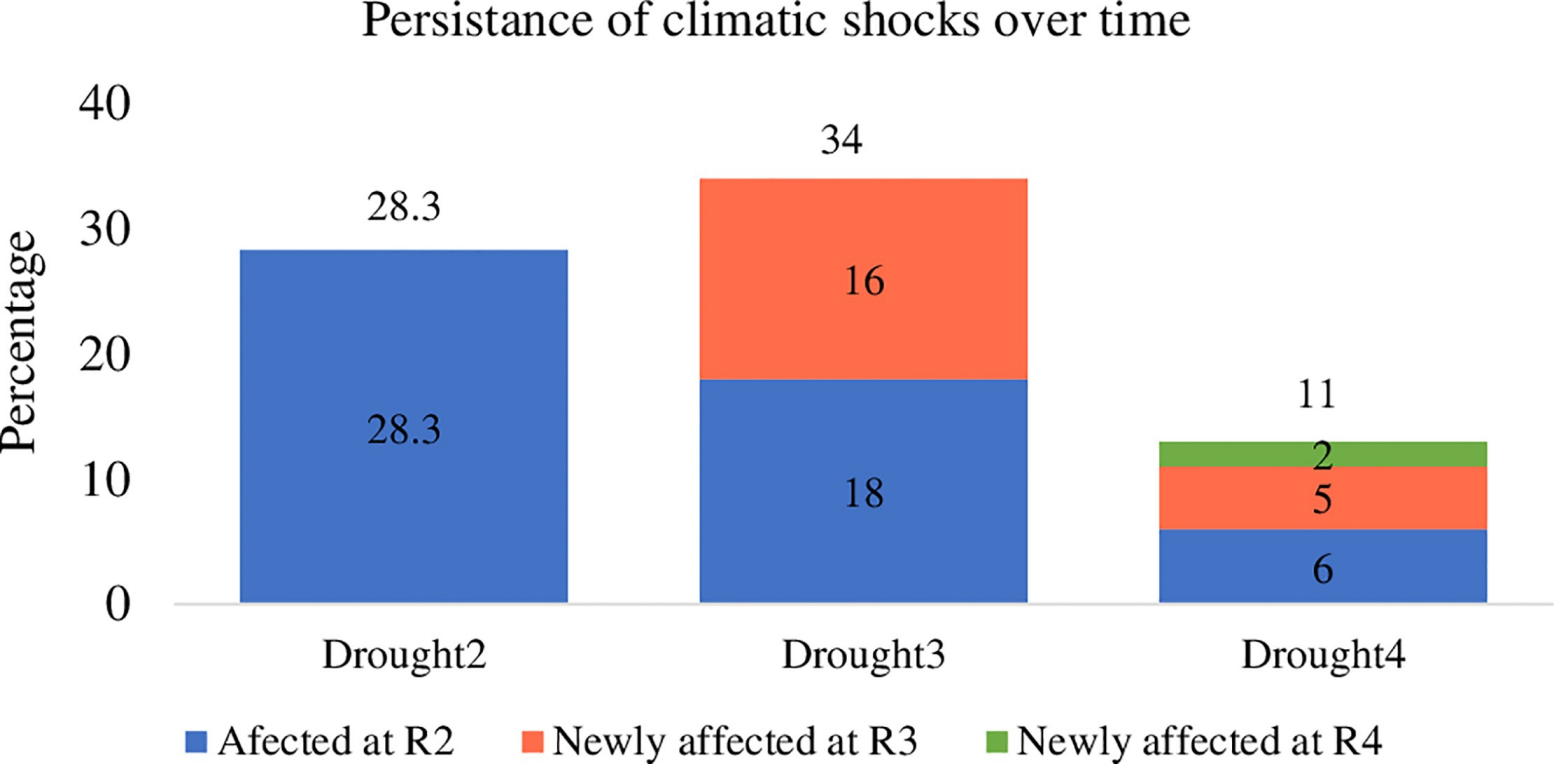
Patterns of drought over time, YL, Ethiopia.

The incidence and prevalence of drought are substantial. The percentage of households reporting drought varies from 34% at round 3 to below 13% in round 4. About 46% of the sample households were affected by drought in either one of the survey rounds and over 6% were affected in all rounds of the survey (Fig 2). Nearly 18% of the households experienced drought in the second and third rounds of the survey, and about 5% experienced drought in the third and fourth rounds of the survey. The proportion of newly affected HHs decreased over time with close to 16% and 2% of the households being newly affected at R3 and R4 respectively.

### Drought and child height-for-age standard score

A paired t-test was conducted to compare HAZ score of children who were affected by drought and those who were not (Table 2). A significant difference in the HAZ score was observed between children who were exposed to drought (-1.28 (1.10)) and those who were not exposed to drought at least in one of the survey rounds (1.62 (1.03)); t(5634) = 10.3310, p<0.000. This suggests that children who are exposed to drought have a lower HAZ score than those who were not. The result reported in Table 2 below is for the pooled data for all rounds. Analysis for each round also shows similar results.

**Table 2:** Paired t-test results of the association of drought and HAZ score.

Group	Obs	Mean	Std. Err.	Std. Dev.	[95% Conf.	Interval]
No	4216	-1.28	0.02	1.1	-1.31	-1.25
Yes	1420	-1.62	0.03	1.03	-1.68	-1.57
Combined	5636	-1.37	0.01	1.09	-1.4	-1.34
diff		0.34	0.03		0.28	0.41
T (Ho: diff = 0)	10.33, p (Ho: diff = 0) = 0					

Degree of freedom = 563.

### Effect of drought on linear growth: Path analysis

The direct, indirect, and total association of exposure to drought and child nutritional status at different stages of the child’s development is presented in Table 3. There are three important results to note. First, concurrent exposure to drought was negatively associated with the HAZ score (*p* < 0.001). Second, early exposure to drought was negatively associated with the HAZ score (*p* < 0.001). Third, growth in interim periods had a mediation role in the pathway through which early life drought exposure transcends to affect nutritional status later in life (*p* < 0.001).

**Table 3:** The direct, indirect, and total association of drought and HAZ score at different stages of the child’s development.

Structural	Direct effect β(SE)		Indirect effect β(SE)	Total effect β(SE)
HAZ 5y	0.210***	0.553***		0.763***
	(0.018)	(0.029)		(0.028)
HAZ 8y	0.554***			0.554***
	(0.018)			(0.018)
HAZ 12y	0.096***	0.312***		0.408***
	(0.009)	(0.009)		(0.011)
Drought 5y	-0.007	-0.058***		-0.065***
	(0.039)	(0.034)		(0.051)
Drought 8y	-0.054***	-0.045***		-0.099***
	(0.038)	(0.022)		(0.044)
Drought 12y	-0.067***			-0.067***
	(0.058)			(0.058)
Sex (Female)	-0.056***			-0.056***
	(0.030)			(0.030)
Child health	0.042***			0.042***
	(0.018)			(0.018)
High SES	0.048			0.048
	(0.062)			(0.062)
PSNP	-0.008			-0.008
	(0.045)			(0.045)
Maternal education	0.009			0.009
	(0.020)			(0.020)
Maternal edu*high SES	-0.065**			-0.065**
	(0.027)			(0.027)
Droughtpsnp4	0.035*			0.035*
	(0.102)			(0.102

a = estimates are adjusted for household food insecurity status, socioeconomic tertile, program participation, maternal education, dependency ratio, types of residence, and availability of public health facility in the community). PSNP = Productive Safety Net Program.

* significant at 10%

** significant at 5%

*** significant at 1%. HAZ 1y, HAZ 5y, HAZ 8y, and HAZ 12y stands for height-for-age z-score ate age 1, 5, 8, and 12 years respectively. Drought 5y, Drought 8y, and Drought 12y refer to drought exposure at 5, 8, and 12 years respectively.

The total association between exposure to drought at the age of 5 years and the HAZ score at the age of 12 years was negative (*p* < 0.001). A large portion of this association was indirect (86%) and mediated through reduction of child growth at the age 8 years, which in turn associated with the HAZ score at age 12 years (*p* < 0.001). Similarly, the total association of drought exposure at age 8 and HAZ score at age 12 years was negative and significant (*p* < 0.001). 56% of this association was direct and 46% of this association was mediated through reduced growth of the child in growing up to 12 years (*p* < 0.001). The total association of exposure to drought at the age of 5 years and the HAZ score at the age of 8 years was negative and significant (*p* < 0.001). The indirect component of this association was mediated through the HAZ score at the age of 5 years (*p* < 0.001). Similarly, the association of drought at the age of 5, 8, and 12 years with the respective HAZ scores at the age of 5, 8, and 12 years was negative and significant. The total association of the HAZ score at age 5 and 12 years was positive (*p* < 0.001). A large proportion of this association was indirect (73%) and mediated through the HAZ score at age 8 years, which in turn was positively associated with the HAZ score at age 12 (*p* < 0.001) (Table 3). Similarly, the HAZ score at the age of 8 years was positively associated with the HAZ score at the age of 12 years (*p* < 0.001) (S2 and S3 Tables).

As far as the role of child, household, and community level covariates is concerned, being female was negatively associated with HAZ score (*p* < 0.001). Good child health status (*p* < 0.001), the existence of a public health facility in the community (*p* = 0.12), PSNP enrolment (*p* = 0.07) and maternal education (*p* > 0.1) were positively associated with the HAZ score. However, for children belonging to households in the highest socio-economic tertile, maternal education had a negative association with the HAZ score (*p* = 0.05). Moreover, households who reported exposure to drought during prior rounds of the survey were more likely to report drought in subsequent rounds (Table 3). Association of other child and household level covariates can be found in the S2 Table.

For robustness check, the data was fitted into Ordinary Least Square, Instrumental Variable Regression, and Fixed Effect model. No major difference in the direction and significance of association was observed except for a slight change in the magnitude of coefficients (S4 Table).

## Discussion

Child chronic undernutrition remains one of the pressing public health concerns in Ethiopia [46–49]. The causes are multifactorial and climate is one of the most important factors [8, 25, 49]. Nonetheless, the literature on the long-term impacts of climatic shocks has largely focused on children under 5 years and largely draws on cross-sectional data. Existing longitudinal studies relied only on two-time points and fixed effect model. The direction and significance of associations vary by the type of anthropometric index, country and extreme weather event under consideration and hence the evidence is inconclusive. This study estimates a path model of the association of exposure to concurrent and long-term exposure to drought at various stages of the child’s development and HAZ scores using four rounds of data from the YL cohort study. Results show that concurrent and long-term exposure to drought is negatively associated with child growth. The impact of exposure to drought at the age of 5 and 8 years on the HAZ score at the age of 12 years is mainly indirect and is mediated through reduced child growth in subsequent periods. Consistently, previous studies have also reported the negative effect of drought on child nutritional status. Using data on under 5 years old children, Alderman et al. [22] have reported that exposure to drought decreases the HAZ score of 12–36 months old Tanzanian children and further reduces height during their adolescence [22].

Several possible explanations can be sought on how drought reduces child growth. First, for a country like Ethiopia, where the majority of the population earns a living from rainfed agriculture[50], drought might affect child nutrition through agricultural pathways. Drought might lead to crop failure, increase food prices, depress economic activity which results in limited access to food and reduces nutrient intake [8, 24–26, 49]. Moreover, as documented in studies from Ethiopia, drought might increase household food insecurity [27, 28, 49] and cause persistent reduction in food consumption [29, 30] and therefore, children in drought-affected households may not able to get sufficient food in subsequent periods to compensate for reduced child growth during the drought period. Second, drought might affect child nutrition through environmental pathways i.e. via a reduction in the availability and quality of drinking water and increase in the occurrence of diseases such as diarrhoea, fever, and acute respiratory infections [8, 31–33]. Exposure to disease and infections, in turn, affects the absorption of nutrients contained in food and might lead to undernutrition [34]. Third, drought might affect child nutrition through income pathways―by increasing the opportunity cost of child health expenditure and leading to lower investment in child health. Moreover, in the absence of functioning credit markets, households might have to liquidate assets. This reduces household’s asset base and future investment in child health and nutrition, and time devoted for child care (e.g. prevent mothers to access and use basic health care services), and introduce time burden for children [31, 35].

The long-term impact of undernutrition during the first 1,000 days and early childhood on physical and mental development has been documented [15, 17, 18]. This evidence has made children in the first 1,000 days window a target for various child nutrition interventions [17]. However, our study showed that the negative impact of drought exposure beyond the 1,000 days window could transcend to affect growth during late childhood and early adolescence. Given the importance of this period for child growth, cognitive achievement, and wellbeing during adulthood children beyond the 1,000 days window should also be a focus of disaster relief programs. Mitigating the impact that drought might have on the physical and mental development at this age group will be the second window of opportunity for "catch-up" growth and breaking intergenerational cycles of undernutrition.

Contrary to other studies from sub-Saharan Africa which documented a higher likelihood of boys to be stunted than girls [51, 52], this study found that being female was negatively associated with HAZ score. This finding is also against the literature that documented that girls have behavioural and biological advantages (e.g. they practice less risky behaviours and have sex hormones that modulate lipid levels and help to boost immune response) [53–55] However, girls are subject to gender and cultural biases which might affect their nutritional status. For instance, studies from Ethiopia have shown that boys receive favouritism in food buffering during food shortages [56]. Ethiopian boys are also favoured in resource allocation for education [57]. Therefore, the negative association of being a girl with HAZ score may signal the existence of negative influence from socio-cultural factors in Ethiopia that outweigh girls behavioural and biological advantages. The difference in the findings as compared to other studies might also be due to the difference in age group considered. While other studies considered children under the age of 5 years, this study considers children between the age of 5 and 12 years.

In this study, PSNP participation by drought-affected households was positively associated with child HAZ score. This implies that safety nets may help smooth the negative impact of drought on child undernutrition. Evidence on the impact of PSNP on child nutrition is mixed. Debela et al. [58] found a positive effect of PSNP participation on child BMI. Berhane et al. [59] on the other hand reported no impact of the program on reducing child undernutrition as measured by HAZ score. The difference might be due to the difference in the sample population, sample size, the types of analysis performed and the anthropometric indices considered in other studies. This calls for further analysis using robust statistical methods, which take into account endogenous selection and time-varying confounding bias.

In our study, maternal education was found to be positively associated with HAZ score. However, the effect of maternal education was negative for children in the highest wealth tertile households. This may be due to the effect that educated mothers are more likely to take up additional productive work, which decreases the time available for childcare. The negative association indicates that the negative effect of compromised care is not outweighed by the positive income effect of increased earnings from maternal education. Our study also revealed households’ who had prior drought exposure were more likely to report drought in subsequent rounds, i.e., the same group of people was repeatedly affected by drought. Therefore, diversifying the means of livelihoods should be considered as a policy alternative for this group of the population.

In conclusion, drought exposure beyond the 1,000 days window, at the age of 5 years, has a long-term impact on child nutrition, at the age of 12 years (i.e. during early adolescence period). The long-term effect of drought on child linear growth was mainly mediated through linear growth during the interim period. Therefore, although evidence on the long-term impact of nutritional impairment in the first 1,000 days window on physical and mental growth and development has made this critical period a focus of various child nutrition intervention programs, our study revealed that exposure to risk factors such as drought after the 1,000 days window might also have a long-lasting impact. Hence, children beyond the 1,000 days window should also be a focus of disaster relief and prevention programs. This might help to prevent the long-term impact of drought on physical and mental growth and to break the intergenerational cycles of undernutrition. Moreover, individual factors such as age and sex, and household level characteristics such as maternal education and enrolment in PSNP by drought-affected households were also positively associated with child linear growth. This signals that participation in safety net programs such as PSNP could help to smooth the impact of drought.

### Strengths and limitations of the study

This study employed a novel approach to analyse the longitudinal association of drought exposure with child nutritional status at different stages of the child’s development. The use of a path model allowed us to analyse the relationship between explanatory and dependent variables at the same time and the decomposition of correlations among variables and hence gave a clear picture of how one variable affects another [21]. Moreover, unlike other studies from the YL dataset [3–5, 13], this study used more rounds of data and conducted a path analysis to investigate the impact of exposure to drought at an early age on nutritional status at later age and the role of interim growth along this pathway. Notwithstanding, our estimates have some methodological limitations: First, in this study, even though drought is a covariate shock by nature, due to the high percentage of intra-cluster variation at child level, the association of drought with child undernutrition was estimated at the child level. Therefore, any generalization from our study of drought as a covariate shock might lead to atomistic fallacy. Second, drought exposure was measured based on the household's self-perception and experience. The level and pattern of drought were not crosschecked using meteorological data. Although self-reported shocks at household level give a good picture of the variation in climatic conditions across different regions within a country and the vulnerability of individual households towards drought, high-quality meteorological data specific to the residence of the households would have been more accurate. Third, although this study tried to control for the impact of drought during the 1,000 days window by introducing the HAZ score of the child at age 1 year, the possibility that children who were affected by drought at age 5 years might have also been affected by drought during the 1,000 days window cannot be ruled out. In addition, although studies have demonstrated the effect of seasonality on child nutrition, our analysis does not take into account the effect of seasonality. Further studies might use meteorological data, examine the effect of intra-household “food buffering” and seasonality, and the endogenous lagged effect of early life (during 1,000 days) drought exposure on growth.

## Supporting information

S1 File Mathematical expression of the path model. (DOCX)

S1 Table Estimates of unstandardized beta coefficients based on full information maximum likelihood and multiple imputation estimations. (DOCX)

S2 Table The direct, indirect and total association of drought and nutritional status at different stages of the child’s development, results of SEM model. (DOCX)

S3 Table Decomposition of indirect effects of earlier exposure to drought on nutritional status at later age. (DOCX)

S4 Table Model robustness check. (DOCX)
