# Correction: Drought and child undernutrition in Ethiopia: A longitudinal path analysis

**DOI:** 10.1371/journal.pone.0220075

**Published:** 2019-07-16

**Authors:** Bezawit Adugna Bahru, Christine Bosch, Regina Birner, Manfred Zeller

## Notice of republication

This article was republished on July 8, 2019 to remove a Supporting Information file that was incorrectly included in the originally published article. The article’s Data Availability statement has also been updated to reflect this change. Please download this article again to view the correct version
