# Supplementary material for: Drought and child undernutrition in Ethiopia: A longitudinal path analysis
Source: PLoS One. 2019 Jun 17;14(6):e0217821. doi: 10.1371/journal.pone.0217821 (PMC6576771; doi:10.1371/journal.pone.0217821)
Supplement: S1 File — (DOCX) [file pone.0217821.s001.docx]

# S1 File. Mathematical expression of the path model

The path diagram in Figure 1 can be alternatively expressed in terms of the following set of equations:

$${HAZ}_{12}=\beta_{0}+\beta_{1}{HAZ}_{8}+\beta_{2}{HAZ}_{5}+\beta_{3}{Drou}_{12}+\beta_{4}{Drou}_{8}+\beta_{5}{Drou}_{5}+u_{12} \left( 1 \right)$$

$${HAZ}_{8}=\alpha_{0}+\alpha_{1}{HAZ}_{5}+\alpha_{2}{Drou}_{8}+{\alpha_{3}{Drou}_{5}+ u}_{8} \left( 2 \right)$$

$${HAZ}_{5}=\gamma_{0}+\gamma_{1}{Drou}_{5}+u_{5} \left( 3 \right)$$

where ${HAZ}_{5}$, ${HAZ}_{8}$, and ${HAZ}_{12}$ refers to HAZ score at age 5, 8, and 12 years respectively. $\beta_{0}$, $\alpha_{0}$, and $\gamma_{0}$ stands for intercepts of regression estimate of ${HAZ}_{5}$, ${HAZ}_{8}$, and ${HAZ}_{12}$ respectively. $\beta_{1}$and $\beta_{2}$ are estimates of the direct effect of ${HAZ}_{5}$ and ${HAZ}_{8}$ respectively on ${HAZ}_{12}$; $\beta_{3}$, $\beta_{4}$, and $\beta_{5}$ captures the direct effect of ${Drou}_{5}$, ${Drou}_{8}$, and ${Drou}_{12}$ respectively on ${HAZ}_{5}$, ${HAZ}_{8}$, and ${HAZ}_{12}$; $\alpha_{1}$, $\alpha_{2},$ and $\alpha_{3}$ are estimates of the direct effect of ${HAZ}_{5}$, ${Drou}_{8}$, and ${Drou}_{5}$ respectively on ${HAZ}_{8}$. $\gamma_{1}$ captures the direct effect of ${Drou}_{5}$ on ${HAZ}_{5}$. $u_{12}u_{8}$, and $u_{5}$ represent factors other than previous HAZ score and drought that affected${HAZ}_{5}$, ${HAZ}_{8}$, and ${HAZ}_{12}$ respectively.

Substituting equation 3 into 2 yields

$${HAZ}_{8}=ϴ_{0}+ϴ_{1}u_{5}+ϴ_{2}{Drou}_{5}+ϴ_{3}{Drou}_{8}+u_{8} \left( 4 \right)$$

Where $ϴ_{0}$= $\alpha_{0}+\alpha_{1}\gamma_{0}$; $ϴ_{1}$= $\alpha_{1}$;and $ϴ_{2}$= $\alpha_{3}$+ $\alpha_{1}\gamma_{1}$; $ϴ_{3}$= $\alpha_{5}$. $ϴ_{0}$ represents the constant term in regression. $ϴ_{1}$captures the direct effect of ${HAZ}_{5}$ on ${HAZ}_{8}$; $ϴ_{2}$captures the direct $(\alpha_{3})$and indirect―through ${HAZ}_{3}$ $(\alpha_{1}*\gamma_{1})$effect of ${Drou}_{5}$ on ${HAZ}_{8}$; $ϴ_{3}$ captures the direct effect of ${Drou}_{8}$ on ${HAZ}_{8}$; and $u_{3}$ captures the effect of other determinants of ${HAZ}_{8}$that are not included in the model.

Substituting equation 4 and 3 into 1 yields

$${HAZ}_{12}=\delta_{0}+\delta_{1}u_{3}+\delta u_{2}+\delta_{3}{Drou}_{5}+\delta_{4}{Drou}_{8}+ {ϴ\delta}_{5}{Drou}_{12}+ u_{4} \left( 5 \right)$$

Where $\delta_{0}$= $\beta_{0}+\beta_{1}\alpha_{0}+\beta_{1}{\alpha_{1}\gamma}_{0}+\beta_{2}\gamma_{0}$; $\delta_{1}$= $\beta_{1}$; $\delta_{2}$= $\beta_{2}$+ ${\alpha_{1}\beta}_{1}$; $\delta_{3}$= $\gamma_{1}\alpha_{1}\beta_{1}$+ ${\alpha_{3}\beta}_{1}$+ ${\gamma_{1}\beta}_{2}$+$\beta_{5}$ ; $\delta_{4}$= ${\alpha_{2}\beta}_{1}$ + $\beta_{4}$; and $\delta_{5}$=$\beta_{3}$

Where $\delta_{0}$ represent the regression coefficient; $\delta_{1}$captures the direct effect of ${HAZ}_{8}$ on ${HAZ}_{12}$; $\delta_{2}$ captures the direct ($\beta_{2}$) and indirect―through ${HAZ}_{8}$ (${\alpha_{1}*\beta}_{1})$ effect of ${HAZ}_{5}$ on ${HAZ}_{12}$; $\delta_{3}$ captures the direct ($\beta_{5}$) and indirect―through ${HAZ}_{5}$ itself (${\gamma_{1}\beta}_{2})$ and through its effect on ${HAZ}_{8}$ ($\gamma_{1}{*\alpha}_{1}{*\beta}_{1})$ of ${Drou}_{5}$ on ${HAZ}_{12}$; $\delta_{4}$ captures the direct ($\beta_{4}$) and indirect―through ${HAZ}_{3}$ (${\alpha_{2}*\beta}_{1})$ effect of ${Drou}_{8}$ on ${HAZ}_{12}$; and $\delta_{5}$ captures the direct effect of ${Drou}_{12}$ on ${HAZ}_{12}$. After adding other potential child, household, and community level covariates, we estimated equation 5 using structural equation modeling. Error terms are assumed to be independent and not correlated with HAZ score in the respective equations for the sake of simplicity of expression of the algebraic form. However, the empirical model accounts of correlation of drought exposure across survey rounds. The final model adjusts for other child, household and community level covariates and other covariates are omitted from the mathematical specification for simplification purpose.
