# Supplementary material for: Drought and child undernutrition in Ethiopia: A longitudinal path analysis
Source: PLoS One. 2019 Jun 17;14(6):e0217821. doi: 10.1371/journal.pone.0217821 (PMC6576771; doi:10.1371/journal.pone.0217821)
Supplement: S1 Table — (DOCX) [file pone.0217821.s002.docx]

# S1 Table. Estimates of unstandardized beta coefficients based on full information maximum likelihood and multiple imputation estimations.

|  | Model 1 | | Model 2 | | Model 3 | |
| --- | --- | --- | --- | --- | --- | --- |
| HAZ 5y | MI | FIML | MI | FIML | MI | FIML |
| HAZ 1y |  |  | 0.235*** | 0.235*** | 0.235*** | 0.235*** |
|  |  |  | (0.012) | (0.01) | (0.012) | (0.01) |
| Drought 5y | -0.357*** | -0.358*** | -0.289*** | -0.289*** | -0.289*** | -0.289*** |
|  | (0.056) | (0.06) | (0.052) | (0.05) | (0.052) | (0.05) |
| _cons | -1.353*** | -1.353*** | -1.012*** | -1.012*** | -1.012*** | -1.012*** |
|  | (0.03) | (0.03) | (0.033) | (0.03) | (0.033) | (0.03) |
| HAZ 8y |  |  |  |  |  |  |
| HAZ 5y | 0.681*** | 0.680*** | 1.014*** | 1.014*** | 1.014*** | 1.013*** |
|  | (0.018) | (0.02) | (0.047) | (0.05) | (0.047) | (0.05) |
| Drought 5y | 0.018 | 0.017 | 0.145*** | 0.144** | 0.144*** | 0.143** |
|  | (0.047) | (0.05) | (0.053) | (0.05) | (0.053) | (0.05) |
| Drought 8y | -0.178*** | -0.178*** | -0.196*** | -0.196*** | -0.195*** | -0.196*** |
|  | (0.045) | (0.04) | (0.044) | (0.04) | (0.044) | (0.04) |
| _cons | -0.170*** | -0.171*** | 0.285*** | 0.284*** | 0.285*** | 0.284*** |
|  | (0.034) | (0.03) | (0.069) | (0.07) | (0.069) | (0.07) |
| HAZ 12y |  |  |  |  |  |  |
| HAZ 5y | 0.209*** | 0.209*** | 0.188*** | 0.188*** | 0.187*** | 0.188*** |
|  | (0.018) | (0.02) | (0.018) | (0.02) | (0.018) | (0.02) |
| HAZ 8y | 0.508*** | 0.508*** | 0.491*** | 0.491*** | 0.491*** | 0.489*** |
|  | (0.018) | (0.02) | (0.018) | (0.02) | (0.018) | (0.02) |
| HAZ 1y |  |  | 0.046*** | 0.046*** | 0.049*** | 0.049*** |
|  |  |  | (0.009) | (0.01) | (0.009) | (0.01) |
| Drought 5y | -0.029 | -0.03 | -0.034 | -0.036 | -0.019 | -0.016 |
|  | (0.037) | (0.04) | (0.037) | (0.04) | (0.039) | (0.04) |
| Drought 8y | -0.121*** | -0.119*** | -0.135*** | -0.134*** | -0.117*** | -0.114** |
|  | (0.035) | (0.04) | (0.035) | (0.04) | (0.038) | (0.04) |
| Drought 12y | -0.167*** | -0.166*** | -0.159*** | -0.158*** | -0.205*** | -0.202*** |
|  | (0.048) | (0.05) | (0.048) | (0.05) | (0.058) | (0.06) |
| Age |  |  | -0.34 | -0.345 | -0.321 | -0.327 |
|  |  |  | (0.266) | (0.27) | (0.265) | (0.26) |
| Age square |  |  | 0.001 | 0.001 | 0.001 | 0.001 |
|  |  |  | (0.001) | (0.001) | (0.001) | (0001) |
| Sex (female) |  |  | -0.111*** | -0.110*** | -0.114*** | -0.112*** |
|  |  |  | (0.03) | (0.03) | (0.03) | (0.03) |
| Child health |  |  | 0.045*** | 0.047** | 0.047*** | 0.050** |
|  |  |  | (0.018) | (0.02) | (0.018) | (0.02) |
| DDS |  |  | -0.01 | -0.009 | -0.009 | -0.009 |
|  |  |  | (0.011) | (0.01) | (0.012) | (0.01) |
| Food secure |  |  |  |  | 0.014 | 0.01 |
|  |  |  |  |  | (0.038) | (0.04) |
| High SES |  |  |  |  | 0.041 | 0.102 |
|  |  |  |  |  | (0.055) | (0.06) |
| PSNP |  |  |  |  | -0.025 | -0.021 |
|  |  |  |  |  | (0.045) | (0.04) |
| Drought*PSNP |  |  |  |  | 0.190* | 0.185 |
|  |  |  |  |  | (0.102) | (0.1) |
| Maternal Education |  |  |  |  | 0.005 | 0.009 |
|  |  |  |  |  | (0.02) | (0.02) |
| Maternaleduc*SES |  |  |  |  | -0.035* | -0.053 |
|  |  |  |  |  | (0.023) | (0.03) |
| Dependency ratio |  |  |  |  | -0.097*** | -0.096** |
|  |  |  |  |  | (0.033) | (0.03) |
| Residence (Rural) |  |  |  |  | -0.012 | 0.007 |
|  |  |  |  |  | (0.043) | (0.04) |
| Public health facility |  |  |  |  | 0.059* | 0.055 |
|  |  |  |  |  | (0.036) | (0.04) |
| _cons | -0.442*** | -0.441*** | 24.161 | 24.483 | 22.669 | 23.061 |
|  | (0.026) | (0.03) | (19.34 | (19.32) | (19.3) | (19.26) |

Model 1=unadjusted, Model 2=adjusted for child characteristics (age, sex, general health status, dietary diversity score, and baseline HAZ), and Model 3=adjusted for household and community characteristics (household food insecurity status, socio economic tertile, program participation, maternal education, dependency ratio, types of residence, and availability of public health facility in the community). MI refers to estimated results from the imputed data and FIML refers to full information maximum likelihood. * significant at 10%, ** significant at 5%, and *** significant at 1%. HAZ 1y, HAZ 5y, HAZ 8y, and HAZ 12y stands for height-for-age z-score ate age 1, 5, 8, and 12 years respectively. Drought 5y, Drought 8y, and Drought 12y refer to drought exposure at 5, 8, and 12 years respectively.Standard errors are given in parentheses.
