# Supplementary material for: Drought and child undernutrition in Ethiopia: A longitudinal path analysis
Source: PLoS One. 2019 Jun 17;14(6):e0217821. doi: 10.1371/journal.pone.0217821 (PMC6576771; doi:10.1371/journal.pone.0217821)
Supplement: S3 Table — (DOCX) [file pone.0217821.s004.docx]

S3 Table. Decomposition of indirect effects of earlier exposure to drought on nutritional status at later age.

| Path | β |
| --- | --- |
| Drought 5y -> HAZ 5y -> HAZ 12y | -0.024*** |
|  | (0.005) |
| Drought 5y -> HAZ 5y -> HAZ 8y -> HAZ 12y | -0.065*** |
|  | (0.012) |
| Drought 5y -> HAZ 8y -> HAZ 12y | 0.032*** |
|  | (0.012) |
| Drought 8y -> HAZ 8y -> HAZ 12y | -0.045*** |
|  | (0.010) |
| HAZ 5y -> HAZ 8y -> HAZ 12y | 0.554*** |
|  | (0.03) |

HAZ 1y, HAZ 5y, HAZ 8y, and HAZ 12y stands for height-for-age z-score ate age 1, 5, 8, and 12 years respectively. Drought 5y, Drought 8y, and Drought 12y refer to drought exposure at 5, 8, and 12 years respectively. β=path coefficient. ${HAZ}_{5}$, ${HAZ}_{8}$, and ${HAZ}_{12}$ stands for height-for-age z-score ate age 5, 8, and 12 years respectively. *significant at 10%, ** significant at 5%, and *** significant at 1%. Standard errors are given in parentheses.
