# Supplementary material for: Drought and child undernutrition in Ethiopia: A longitudinal path analysis
Source: PLoS One. 2019 Jun 17;14(6):e0217821. doi: 10.1371/journal.pone.0217821 (PMC6576771; doi:10.1371/journal.pone.0217821)
Supplement: S4 Table — (DOCX) [file pone.0217821.s005.docx]

# S4 Table. Model robustness check.

| HAZ 12y | SEM^1^ | OLS^2^ | ivreg | Ivreg^3^ | FE^4^ | Xtivreg^5^ | xtivregt |
| --- | --- | --- | --- | --- | --- | --- | --- |
| Drought 5y | -0.065*** | -0.285***  (0.058) | -1.543***  (0.571) | -3.612**  (1.412) | -0.045  (0.056) | 0.265  (0.741) | 0.265 |
|  | (0.051) |  |  |  |  |  | (0.741) |
| Drought 8y | -0.099*** |  |  |  |  |  |  |
|  | (0.044) |  |  |  |  |  |  |
| Drought 12y | -0.067*** |  |  |  |  |  |  |
|  | (0.058) |  |  |  |  |  |  |
| Age | -1.273 | 0.005 | 0.006 | -0.018 | 0.006 | 0.011 | 0.011 |
|  | (0.265) | (0.025) | (0.027) | (0.028) | (0.016) | (0.015) | (0.015) |
| Age square | 1.282 | -0.000 | -0.000 | 0.000 | -0.000 | -0.000 | -0.000 |
|  | (0.001) | (0.000) | (0.000) | (0.000) | (0.000) | (0.000) | (0.000) |
| Sex (female) | -0.056*** | -0.015 | -0.026 | -0.006 |  | -0.014 | -0.014 |
|  | (0.03) | (0.037) | (0.042) | (0.039) |  | (0.049) | (0.049) |
| HAZ 5y | 0.408*** | 0.205*** | 0.204*** | 0.204*** |  | 0.210*** | 0.210*** |
|  | (0.011 | (0.009) | (0.010) | (0.010) |  | (0.012) | (0.012) |
| Child health | 0.042*** | 0.059*** | 0.044* | 0.061*** |  | 0.065** | 0.065** |
|  | (0.018) | (0.023) | (0.025) | (0.023) |  | (0.030) | (0.030) |
| DDS | -0.012 | 0.004 | -0.031 | -0.005 | -0.007 | -0.000 | -0.000 |
|  | (0.012) | (0.014) | (0.022) | (0.015) | (0.013) | (0.011) | (0.011) |
| Food insecure | 0.004 | -0.016 | 0.037 | -0.082 | -0.111** | -0.083* | -0.083* |
|  | (0.038) | (0.052) | (0.061) | (0.062) | (0.048) | (0.043) | (0.043) |
| High SES | 0.048 | 0.416*** | 0.337*** | 0.392*** | -0.102 | 0.164** | 0.164** |
|  | (0.062) | (0.077) | (0.092) | (0.080) | (0.103) | (0.072) | (0.072) |
| PSNP | -0.008 | -0.171*** | -0.605*** | -0.224*** | -0.001 | -0.096* | -0.096* |
|  | (0.045) | (0.055) | (0.203) | (0.060) | (0.067) | (0.051) | (0.051) |
| Maternal educ | 0.009 | 0.085*** | 0.102*** | 0.095*** | -0.084 | 0.083*** | 0.083*** |
|  | (0.02) | (0.024) | (0.027) | (0.025) | (0.234) | (0.030) | (0.030) |
| Maternal educ*SES | -0.065** | -0.068* | -0.097** | -0.085** | 0.038 | -0.007 | -0.007 |
|  | (0.027) | (0.036) | (0.042) | (0.038) | (0.063) | (0.038) | (0.038) |
| Dependency ratio | -0.045*** | -0.103*** | -0.088** | -0.101*** | -0.127** | -0.138*** | -0.138*** |
|  | (0.033) | (0.038) | (0.042) | (0.039) | (0.054) | (0.037) | (0.037) |
| Residence (rural) | 0.003 | 0.149*** | 0.405*** | 0.167*** | 0.646** | 0.005 | 0.005 |
|  | (0.043) | (0.053) | (0.130) | (0.055) | (0.325) | (0.063) | (0.063) |
| Public health facility | 0.027 | 0.072* | -0.039 | 0.086** | -0.050 | 0.023 | 0.023 |
|  | (0.036) | (0.038) | (0.065) | (0.040) | (0.045) | (0.034) | (0.034) |
| Drought*PSNP | 0.035* | 0.264*** | 1.402*** | 0.474*** | 0.113 | 0.174** | 0.174** |
|  | (0.102) | (0.087) | (0.521) | (0.124) | (0.076) | (0.086) | (0.086) |
| Drought * Time |  |  |  | 0.946** |  | -0.122 | -0.122 |
|  |  |  |  | (0.402) |  | (0.209) | (0.209) |
| Constant |  | -1.554 | -1.242 | 0.215 | -2.219** | -1.603* | -1.603* |
|  |  | (1.459) | (1.609) | (1.690) | (1.099) | (0.946) | (0.946) |
| R-squared |  | 0.236 | 0.086 | 0.196 | 0.090 |  |  |
| N |  | 2414 | 2400 | 2400 | 2414 | 2400 | 2400 |
| F |  | 46.33 | 37.45 | 41.26 | 7.479 | . | . |

1=Structural Equation Model. 2=Ordinary Least Square. 3=Instrumental Variable Regression on the pooled sample using household access to credit, use of any methods to increase yield such as /improved variety or seeds, and use of irrigation. 4=Child Fixed Effects, 5=Instrumental variable Regression on the panel data structure sample using household access to credit, use of any methods to increase yield such as /improved variety or seeds, and use of irrigation, ¥= Estimates show the impact of drought for the panel model rather than the separate impact of drought at different period. HAZ 1y, HAZ 5y, HAZ 8y, and HAZ 12y stands for height-for-age z-score ate age 1, 5, 8, and 12 years respectively. Drought 5y, Drought 8y, and Drought 12y refer to drought exposure at 5, 8, and 12 years respectively. Dependent variable is HAZ score at age 12 for the SEM model and HAZ score for the other models. * significant at 10%, ** significant at 5%, and *** significant at 1%.
